# Bis[*N*′-(2-oxo-1*H*-indol-3-ylidene)furan-2-carbohydrazidato-κ^3^ *O*,*N*′,*O*′]manganese(II) *N*,*N*-dimethyl­formide monosolvate monohydrate

**DOI:** 10.1107/S1600536810039516

**Published:** 2010-10-23

**Authors:** Siti Nadiah Abdul Halim, Hapipah Mohd Ali, Seik Weng Ng

**Affiliations:** a Department of Chemistry, University of Malaya, 50603 Kuala Lumpur, Malaysia

## Abstract

In the title compound, [Mn(C_13_H_8_N_3_O_3_)_2_]·C_3_H_7_NO·H_2_O, the metal atom is *O*,*N*,*O*′-chelated by two deprotonated Schiff bases and exists in a distorted octa­hedral geometry. The N–H groups, the carbonyl group of the DMF mol­ecule and the uncoord­inated water mol­ecule engage in N—H⋯O and O—H⋯O hydrogen-bonding inter­actions, generating a hydrogen-bonded ribbon that propagates along [110].

## Related literature

For the crystal structure of the uncoordinated Schiff base ligand, see: Rodríguez-Argüelles *et al.* (2009 ▶).
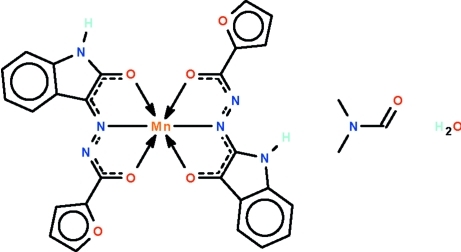


## Experimental

### 

#### Crystal data

[Mn(C_13_H_8_N_3_O_3_)_2_]·C_3_H_7_NO·H_2_O
*M* *_r_* = 654.50
Triclinic, 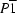

*a* = 11.4833 (7) Å
*b* = 11.5599 (7) Å
*c* = 13.1619 (8) Å
α = 107.580 (1)°
β = 97.800 (1)°
γ = 115.159 (1)°
*V* = 1435.21 (15) Å^3^
*Z* = 2
Mo *K*α radiation
μ = 0.53 mm^−1^
*T* = 295 K
0.45 × 0.32 × 0.20 mm

#### Data collection

Bruker SMART area-detector diffractometer
Absorption correction: multi-scan (*SADABS*; Sheldrick, 1996 ▶) *T*_min_ = 0.798, *T*_max_ = 0.902
12170 measured reflections
6180 independent reflections
4297 reflections with *I* > 2*I*)
*R*_int_ = 0.019

#### Refinement

*R*[*F* ^2^ > 2σ(*F* ^2^)] = 0.041
*wR*(*F* ^2^) = 0.126
*S* = 0.95
6180 reflections
420 parameters
4 restraints
H atoms treated by a mixture of independent and constrained refinement
Δρ_max_ = 0.61 e Å^−3^
Δρ_min_ = −0.26 e Å^−3^

### 

Data collection: *SMART* (Bruker, 2001 ▶); cell refinement: *SAINT* (Bruker, 2001 ▶); data reduction: *SAINT*; program(s) used to solve structure: *SHELXS97* (Sheldrick, 2008 ▶); program(s) used to refine structure: *SHELXL97* (Sheldrick, 2008 ▶); molecular graphics: *X-SEED* (Barbour, 2001 ▶); software used to prepare material for publication: *publCIF* (Westrip, 2010 ▶).

## Supplementary Material

Crystal structure: contains datablocks global, I. DOI: 10.1107/S1600536810039516/ci5164sup1.cif

Structure factors: contains datablocks I. DOI: 10.1107/S1600536810039516/ci5164Isup2.hkl

Additional supplementary materials:  crystallographic information; 3D view; checkCIF report

## Figures and Tables

**Table 1:** Selected bond lengths (Å)

Mn1—N2	2.016 (2)
Mn1—N5	2.023 (2)
Mn1—O5	2.0667 (18)
Mn1—O2	2.076 (2)
Mn1—O3	2.2676 (19)
Mn1—O6	2.2998 (19)

**Table 2:** Hydrogen-bond geometry (Å, °)

*D*—H⋯*A*	*D*—H	H⋯*A*	*D*⋯*A*	*D*—H⋯*A*
O1*W*—H1*W*1⋯O3	0.86 (1)	2.06 (2)	2.896 (3)	165 (4)
O1*W*—H1*W*2⋯O7	0.86 (1)	1.93 (2)	2.762 (5)	161 (4)
N3—H3⋯O1*W*^i^	0.86 (1)	1.96 (1)	2.811 (4)	176 (3)
N6—H6⋯O6^ii^	0.87 (1)	2.05 (1)	2.899 (3)	167 (3)

Symmetry codes: (i) 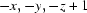; (ii) 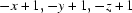.

**Fig. 1.**
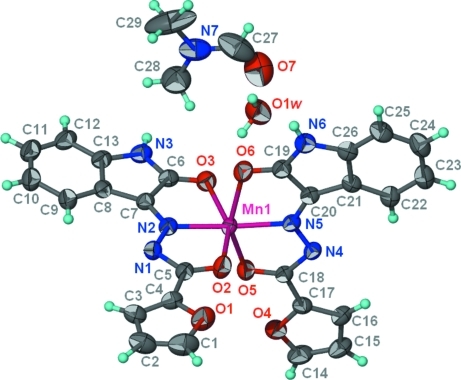
The asymmetric unit of the title compound. Displacement ellipsoids are drawn at the 50% probability level. H atoms are shown as spheres of arbitrary radius.

## supplementary crystallographic information

### Comment

Divalent cobalt, nickel, copper and zinc derivatives of Schiff base condensation product of isatin and 2-furoic acid hydrazide have been synthesized, and these are reported along with the crystal structure of the Schiff base (Rodríguez-Argüelles *et al.*, 2009). If the deprotonated Schiff base is to chelate in a terdentate manner, the anion has to rotate about the nitrogen-nitrogen bond. Such a rotation is observed in the manganese derivative, which crystallizes from DMF as a monohydrated monosolvate (Scheme I). The divalent metal atom is *O*,*N*,*O*'-chelated by two deprotonated Schiff base in an octahedral geometry (Fig. 1). The amino group, the carbonyl group of the DMF and the lattice water molecule engage in N—H···O and O—H···O hydrogen bonding interactions to generate a hydrogen-bonded ribbon that propagates along [110].

### Experimental

The Schiff base was synthesized by condensing isatin and furoylhydrazine according to a literature procedure (Rodríguez-Argüelles *et al.*, 2009). Manganese acetate (1 mmol) and the Schiff base (2 mmol) were heated in ethanol (100 ml) for 5 h; several drops of triethylamine were added. The solvent was removed and the product purified by recrystallization from DMF.

### Refinement

The amino and water H-atoms were located in a difference Fourier map, and were refined with a distance restraint of N–H/O–H = 0.85 (1) Å; their U_iso_ parameters were freely refined. Carbon-bound H-atoms were placed in calculated positions (C–H = 0.93–0.96 Å) and were included in the refinement in the riding model approximation, with *U_iso_*(H) set to 1.2-1.5*U_eq_*(C). The highest peak and deepest hole in the final difference map are located 0.65 Å from Mn1 and 0.57 Å from H28A, respectively.

### Crystal data

**Table:**

[Mn(C_13_H_8_N_3_O_3_)_2_]·C_3_H_7_NO·H_2_O	*Z* = 2
*M**_r_* = 654.50	*F*(000) = 674
Triclinic, *P*1	*D*_x_ = 1.515 Mg m^−^^3^
Hall symbol: -P 1	Mo *K*α radiation, λ = 0.71073 Å
*a* = 11.4833 (7) Å	Cell parameters from 4583 reflections
*b* = 11.5599 (7) Å	θ = 2.2–26.7°
*c* = 13.1619 (8) Å	µ = 0.53 mm^−^^1^
α = 107.580 (1)°	*T* = 295 K
β = 97.800 (1)°	Block, black
γ = 115.159 (1)°	0.45 × 0.32 × 0.20 mm
*V* = 1435.21 (15) Å^3^	

### Data collection

**Table:**

Bruker SMART area-detector diffractometer	6180 independent reflections
Radiation source: fine-focus sealed tube	4297 reflections with *I* > 2˘*I*)
graphite	*R*_int_ = 0.019
ω scans	θ_max_ = 27.1°, θ_min_ = 2.1°
Absorption correction: multi-scan (*SADABS*; Sheldrick, 1996)	*h* = −14→14
*T*_min_ = 0.798, *T*_max_ = 0.902	*k* = −14→14
12170 measured reflections	*l* = −16→16

### Refinement

**Table:**

Refinement on *F*^2^	Primary atom site location: structure-invariant direct methods
Least-squares matrix: full	Secondary atom site location: difference Fourier map
*R*[*F*^2^ > 2σ(*F*^2^)] = 0.041	Hydrogen site location: inferred from neighbouring sites
*wR*(*F*^2^) = 0.126	H atoms treated by a mixture of independent and constrained refinement
*S* = 0.95	*w* = 1/[σ^2^(*F*_o_^2^) + (0.0671*P*)^2^ + 0.6988*P*] where *P* = (*F*_o_^2^ + 2*F*_c_^2^)/3
6180 reflections	(Δ/σ)_max_ = 0.001
420 parameters	Δρ_max_ = 0.61 e Å^−^^3^
4 restraints	Δρ_min_ = −0.26 e Å^−^^3^

### Special details

**Table:**

Geometry. All esds (except the esd in the dihedral angle between two l.s. planes) are estimated using the full covariance matrix. The cell esds are taken into account individually in the estimation of esds in distances, angles and torsion angles; correlations between esds in cell parameters are only used when they are defined by crystal symmetry. An approximate (isotropic) treatment of cell esds is used for estimating esds involving l.s. planes.

### Fractional atomic coordinates and isotropic or equivalent isotropic displacement parameters (Å^2^)

**Table:**

	*x*	*y*	*z*	*U*_iso_*/*U*_eq_	
Mn1	0.48864 (4)	0.33824 (4)	0.74033 (3)	0.03787 (13)	
O1	0.8779 (2)	0.7680 (2)	0.9910 (2)	0.0715 (6)	
O2	0.66584 (18)	0.5205 (2)	0.84440 (17)	0.0580 (5)	
O3	0.26092 (19)	0.2002 (2)	0.67083 (16)	0.0536 (5)	
O4	0.5776 (2)	0.0564 (2)	0.90415 (16)	0.0584 (5)	
O5	0.51399 (19)	0.21270 (19)	0.81422 (15)	0.0510 (5)	
O6	0.4817 (2)	0.4103 (2)	0.59584 (16)	0.0565 (5)	
O7	0.2100 (4)	0.2105 (4)	0.3397 (3)	0.1265 (12)	
O1W	0.1448 (3)	0.0178 (3)	0.4351 (2)	0.0872 (8)	
H1W1	0.168 (4)	0.059 (4)	0.5069 (9)	0.105*	
H1W2	0.153 (4)	0.084 (3)	0.413 (3)	0.105*	
N1	0.5167 (2)	0.5882 (2)	0.90994 (18)	0.0448 (5)	
N2	0.4257 (2)	0.4610 (2)	0.83192 (18)	0.0429 (5)	
N3	0.0846 (2)	0.2401 (3)	0.7046 (2)	0.0530 (6)	
H3	0.017 (2)	0.1608 (18)	0.660 (2)	0.064*	
N4	0.6060 (2)	0.1576 (2)	0.67171 (17)	0.0406 (5)	
N5	0.5641 (2)	0.2372 (2)	0.64017 (17)	0.0406 (5)	
N6	0.5591 (2)	0.3727 (3)	0.4406 (2)	0.0527 (6)	
H6	0.544 (3)	0.430 (3)	0.418 (2)	0.063*	
N7	0.1231 (3)	0.3539 (4)	0.3902 (3)	0.0870 (10)	
C1	0.9606 (4)	0.8932 (4)	1.0792 (3)	0.0838 (11)	
H1	1.0543	0.9406	1.0972	0.101*	
C2	0.8913 (4)	0.9391 (4)	1.1360 (3)	0.0743 (10)	
H2	0.9261	1.0217	1.1989	0.089*	
C3	0.7542 (3)	0.8375 (3)	1.0817 (2)	0.0543 (7)	
H3A	0.6805	0.8401	1.1019	0.065*	
C4	0.7501 (3)	0.7363 (3)	0.9952 (2)	0.0473 (6)	
C5	0.6409 (3)	0.6055 (3)	0.9102 (2)	0.0425 (6)	
C6	0.2148 (3)	0.2725 (3)	0.7204 (2)	0.0456 (6)	
C7	0.2962 (2)	0.4144 (3)	0.8083 (2)	0.0421 (6)	
C8	0.2058 (3)	0.4627 (3)	0.8430 (2)	0.0447 (6)	
C9	0.2232 (3)	0.5855 (3)	0.9194 (2)	0.0553 (7)	
H9	0.3090	0.6589	0.9636	0.066*	
C10	0.1083 (4)	0.5958 (4)	0.9281 (3)	0.0682 (9)	
H10	0.1169	0.6778	0.9779	0.082*	
C11	−0.0186 (3)	0.4850 (4)	0.8634 (3)	0.0709 (9)	
H11	−0.0939	0.4938	0.8718	0.085*	
C12	−0.0379 (3)	0.3611 (4)	0.7864 (3)	0.0623 (8)	
H12	−0.1239	0.2875	0.7430	0.075*	
C13	0.0758 (3)	0.3521 (3)	0.7773 (2)	0.0487 (6)	
C14	0.6181 (3)	−0.0311 (4)	0.9250 (3)	0.0682 (9)	
H14	0.6078	−0.0587	0.9841	0.082*	
C15	0.6745 (3)	−0.0724 (3)	0.8495 (3)	0.0627 (8)	
H15	0.7100	−0.1317	0.8469	0.075*	
C16	0.6695 (3)	−0.0079 (3)	0.7746 (3)	0.0524 (7)	
H16	0.7009	−0.0168	0.7127	0.063*	
C17	0.6104 (3)	0.0691 (3)	0.8102 (2)	0.0441 (6)	
C18	0.5727 (2)	0.1531 (3)	0.7664 (2)	0.0397 (5)	
C19	0.5330 (3)	0.3554 (3)	0.5333 (2)	0.0459 (6)	
C20	0.5793 (2)	0.2607 (3)	0.5509 (2)	0.0422 (6)	
C21	0.6341 (2)	0.2229 (3)	0.4619 (2)	0.0436 (6)	
C22	0.6909 (3)	0.1385 (3)	0.4341 (2)	0.0526 (7)	
H22	0.7013	0.0916	0.4779	0.063*	
C23	0.7322 (3)	0.1256 (3)	0.3391 (3)	0.0636 (8)	
H23	0.7696	0.0683	0.3182	0.076*	
C24	0.7183 (3)	0.1973 (3)	0.2746 (3)	0.0660 (9)	
H24	0.7475	0.1876	0.2116	0.079*	
C25	0.6621 (3)	0.2828 (3)	0.3020 (2)	0.0600 (8)	
H25	0.6531	0.3307	0.2588	0.072*	
C26	0.6202 (3)	0.2940 (3)	0.3953 (2)	0.0479 (6)	
C27	0.1768 (6)	0.2935 (6)	0.3242 (4)	0.121 (2)	
H27	0.1900	0.3152	0.2627	0.145*	
C28	0.1071 (5)	0.3253 (5)	0.4865 (4)	0.1004 (13)	
H28A	0.1883	0.3317	0.5249	0.151*	
H28B	0.0329	0.2329	0.4643	0.151*	
H28C	0.0892	0.3919	0.5356	0.151*	
C29	0.0825 (5)	0.4465 (6)	0.3654 (6)	0.167 (3)	
H29A	0.1070	0.4600	0.3013	0.251*	
H29B	0.1269	0.5348	0.4286	0.251*	
H29C	−0.0137	0.4067	0.3498	0.251*	

### Atomic displacement parameters (Å^2^)

**Table:**

	*U*^11^	*U*^22^	*U*^33^	*U*^12^	*U*^13^	*U*^23^
Mn1	0.0398 (2)	0.0382 (2)	0.0430 (2)	0.02413 (17)	0.01540 (16)	0.01709 (17)
O1	0.0411 (11)	0.0745 (15)	0.0855 (16)	0.0201 (11)	0.0176 (11)	0.0284 (13)
O2	0.0464 (11)	0.0552 (12)	0.0704 (13)	0.0277 (10)	0.0209 (10)	0.0174 (10)
O3	0.0535 (11)	0.0492 (11)	0.0541 (11)	0.0263 (10)	0.0129 (9)	0.0159 (9)
O4	0.0677 (13)	0.0746 (14)	0.0528 (11)	0.0411 (11)	0.0250 (10)	0.0384 (11)
O5	0.0597 (12)	0.0580 (12)	0.0510 (11)	0.0368 (10)	0.0260 (9)	0.0261 (9)
O6	0.0698 (13)	0.0656 (13)	0.0609 (12)	0.0460 (11)	0.0292 (10)	0.0358 (10)
O7	0.142 (3)	0.114 (3)	0.128 (3)	0.064 (3)	0.058 (2)	0.046 (2)
O1W	0.0866 (18)	0.0680 (17)	0.0727 (16)	0.0291 (15)	0.0095 (15)	0.0053 (14)
N1	0.0402 (12)	0.0448 (12)	0.0470 (12)	0.0196 (10)	0.0124 (10)	0.0178 (10)
N2	0.0416 (12)	0.0447 (12)	0.0466 (12)	0.0214 (10)	0.0145 (10)	0.0229 (10)
N3	0.0407 (13)	0.0551 (15)	0.0554 (14)	0.0178 (11)	0.0092 (11)	0.0235 (12)
N4	0.0418 (11)	0.0421 (11)	0.0465 (12)	0.0237 (10)	0.0160 (10)	0.0231 (10)
N5	0.0397 (11)	0.0404 (11)	0.0451 (12)	0.0202 (9)	0.0126 (9)	0.0210 (10)
N6	0.0615 (15)	0.0576 (15)	0.0560 (14)	0.0335 (13)	0.0234 (12)	0.0355 (12)
N7	0.079 (2)	0.084 (2)	0.085 (2)	0.0233 (18)	0.0087 (18)	0.049 (2)
C1	0.0438 (18)	0.078 (3)	0.087 (3)	0.0036 (18)	−0.0034 (18)	0.030 (2)
C2	0.069 (2)	0.066 (2)	0.0570 (19)	0.0151 (19)	0.0051 (17)	0.0179 (17)
C3	0.0555 (17)	0.0529 (17)	0.0463 (15)	0.0199 (14)	0.0113 (13)	0.0217 (14)
C4	0.0381 (14)	0.0519 (16)	0.0537 (16)	0.0186 (13)	0.0130 (12)	0.0293 (14)
C5	0.0421 (14)	0.0472 (15)	0.0461 (14)	0.0236 (12)	0.0169 (12)	0.0247 (12)
C6	0.0444 (15)	0.0486 (15)	0.0451 (14)	0.0218 (13)	0.0115 (12)	0.0232 (12)
C7	0.0395 (14)	0.0490 (15)	0.0429 (14)	0.0227 (12)	0.0142 (11)	0.0230 (12)
C8	0.0444 (14)	0.0575 (16)	0.0436 (14)	0.0278 (13)	0.0202 (12)	0.0277 (13)
C9	0.0586 (18)	0.0660 (19)	0.0513 (16)	0.0362 (16)	0.0243 (14)	0.0247 (15)
C10	0.078 (2)	0.091 (3)	0.065 (2)	0.057 (2)	0.0374 (18)	0.0368 (19)
C11	0.066 (2)	0.111 (3)	0.074 (2)	0.060 (2)	0.0384 (19)	0.053 (2)
C12	0.0468 (17)	0.091 (2)	0.068 (2)	0.0381 (17)	0.0243 (15)	0.0469 (19)
C13	0.0437 (15)	0.0616 (17)	0.0506 (15)	0.0265 (14)	0.0180 (12)	0.0323 (14)
C14	0.072 (2)	0.089 (2)	0.072 (2)	0.046 (2)	0.0239 (18)	0.057 (2)
C15	0.0578 (18)	0.071 (2)	0.083 (2)	0.0394 (17)	0.0218 (17)	0.0483 (18)
C16	0.0476 (15)	0.0591 (17)	0.0627 (17)	0.0307 (14)	0.0193 (13)	0.0321 (15)
C17	0.0415 (14)	0.0490 (15)	0.0423 (14)	0.0198 (12)	0.0122 (11)	0.0231 (12)
C18	0.0350 (13)	0.0412 (13)	0.0425 (13)	0.0183 (11)	0.0104 (11)	0.0173 (11)
C19	0.0464 (15)	0.0496 (15)	0.0485 (15)	0.0245 (13)	0.0168 (12)	0.0261 (13)
C20	0.0407 (14)	0.0415 (14)	0.0453 (14)	0.0186 (12)	0.0126 (11)	0.0215 (12)
C21	0.0404 (14)	0.0412 (14)	0.0419 (14)	0.0147 (12)	0.0107 (11)	0.0164 (11)
C22	0.0488 (16)	0.0487 (16)	0.0565 (17)	0.0224 (13)	0.0163 (13)	0.0184 (13)
C23	0.0587 (19)	0.0578 (19)	0.0639 (19)	0.0257 (16)	0.0237 (16)	0.0138 (16)
C24	0.063 (2)	0.067 (2)	0.0546 (18)	0.0222 (17)	0.0278 (16)	0.0187 (16)
C25	0.0611 (19)	0.0615 (19)	0.0511 (17)	0.0201 (16)	0.0219 (15)	0.0280 (15)
C26	0.0439 (15)	0.0442 (15)	0.0494 (15)	0.0154 (12)	0.0137 (12)	0.0205 (12)
C27	0.110 (4)	0.114 (4)	0.074 (3)	0.001 (3)	0.023 (3)	0.038 (3)
C28	0.109 (3)	0.104 (3)	0.104 (3)	0.054 (3)	0.036 (3)	0.057 (3)
C29	0.112 (4)	0.115 (4)	0.229 (7)	0.015 (3)	−0.044 (4)	0.113 (5)

### Geometric parameters (Å, °)

**Table:**

Mn1—N2	2.016 (2)	C6—C7	1.470 (4)
Mn1—N5	2.023 (2)	C7—C8	1.437 (3)
Mn1—O5	2.0667 (18)	C8—C9	1.381 (4)
Mn1—O2	2.076 (2)	C8—C13	1.404 (4)
Mn1—O3	2.2676 (19)	C9—C10	1.391 (4)
Mn1—O6	2.2998 (19)	C9—H9	0.93
O1—C1	1.364 (4)	C10—C11	1.381 (5)
O1—C4	1.367 (3)	C10—H10	0.93
O2—C5	1.261 (3)	C11—C12	1.385 (5)
O3—C6	1.243 (3)	C11—H11	0.93
O4—C14	1.359 (4)	C12—C13	1.371 (4)
O4—C17	1.372 (3)	C12—H12	0.93
O5—C18	1.255 (3)	C14—C15	1.336 (5)
O6—C19	1.249 (3)	C14—H14	0.93
O7—C27	1.231 (7)	C15—C16	1.413 (4)
O1W—H1W1	0.86 (1)	C15—H15	0.93
O1W—H1W2	0.86 (1)	C16—C17	1.350 (4)
N1—N2	1.341 (3)	C16—H16	0.93
N1—C5	1.353 (3)	C17—C18	1.446 (3)
N2—C7	1.301 (3)	C19—C20	1.466 (4)
N3—C6	1.346 (3)	C20—C21	1.443 (4)
N3—C13	1.411 (4)	C21—C22	1.379 (4)
N3—H3	0.86 (1)	C21—C26	1.407 (4)
N4—N5	1.343 (3)	C22—C23	1.385 (4)
N4—C18	1.361 (3)	C22—H22	0.93
N5—C20	1.301 (3)	C23—C24	1.391 (5)
N6—C19	1.348 (3)	C23—H23	0.93
N6—C26	1.415 (4)	C24—C25	1.383 (4)
N6—H6	0.87 (1)	C24—H24	0.93
N7—C27	1.330 (7)	C25—C26	1.369 (4)
N7—C28	1.419 (5)	C25—H25	0.93
N7—C29	1.438 (5)	C27—H27	0.93
C1—C2	1.325 (5)	C28—H28A	0.96
C1—H1	0.93	C28—H28B	0.96
C2—C3	1.408 (4)	C28—H28C	0.96
C2—H2	0.93	C29—H29A	0.96
C3—C4	1.342 (4)	C29—H29B	0.96
C3—H3A	0.93	C29—H29C	0.96
C4—C5	1.448 (4)		
			
N2—Mn1—N5	171.59 (8)	C11—C10—C9	120.4 (3)
N2—Mn1—O5	111.99 (8)	C11—C10—H10	119.8
N5—Mn1—O5	75.50 (7)	C9—C10—H10	119.8
N2—Mn1—O2	75.65 (8)	C10—C11—C12	122.4 (3)
N5—Mn1—O2	100.19 (8)	C10—C11—H11	118.8
O5—Mn1—O2	96.41 (8)	C12—C11—H11	118.8
N2—Mn1—O3	78.39 (8)	C13—C12—C11	116.9 (3)
N5—Mn1—O3	105.49 (8)	C13—C12—H12	121.6
O5—Mn1—O3	93.69 (7)	C11—C12—H12	121.6
O2—Mn1—O3	154.03 (7)	C12—C13—C8	121.7 (3)
N2—Mn1—O6	95.06 (8)	C12—C13—N3	128.3 (3)
N5—Mn1—O6	77.73 (7)	C8—C13—N3	110.0 (2)
O5—Mn1—O6	152.77 (7)	C15—C14—O4	111.3 (3)
O2—Mn1—O6	93.09 (8)	C15—C14—H14	124.3
O3—Mn1—O6	88.68 (7)	O4—C14—H14	124.3
C1—O1—C4	104.9 (3)	C14—C15—C16	106.3 (3)
C5—O2—Mn1	110.94 (16)	C14—C15—H15	126.9
C6—O3—Mn1	105.12 (16)	C16—C15—H15	126.9
C14—O4—C17	105.7 (2)	C17—C16—C15	106.8 (3)
C18—O5—Mn1	111.41 (15)	C17—C16—H16	126.6
C19—O6—Mn1	105.01 (15)	C15—C16—H16	126.6
H1W1—O1W—H1W2	103 (4)	C16—C17—O4	109.9 (2)
N2—N1—C5	107.4 (2)	C16—C17—C18	132.7 (2)
C7—N2—N1	122.6 (2)	O4—C17—C18	117.3 (2)
C7—N2—Mn1	117.46 (18)	O5—C18—N4	125.9 (2)
N1—N2—Mn1	119.86 (16)	O5—C18—C17	120.5 (2)
C6—N3—C13	110.2 (2)	N4—C18—C17	113.6 (2)
C6—N3—H3	124 (2)	O6—C19—N6	127.8 (2)
C13—N3—H3	125 (2)	O6—C19—C20	125.0 (2)
N5—N4—C18	107.04 (19)	N6—C19—C20	107.2 (2)
C20—N5—N4	122.4 (2)	N5—C20—C21	138.0 (2)
C20—N5—Mn1	117.93 (17)	N5—C20—C19	114.3 (2)
N4—N5—Mn1	119.52 (15)	C21—C20—C19	107.7 (2)
C19—N6—C26	110.0 (2)	C22—C21—C26	120.3 (2)
C19—N6—H6	122 (2)	C22—C21—C20	134.3 (2)
C26—N6—H6	128 (2)	C26—C21—C20	105.4 (2)
C27—N7—C28	118.5 (4)	C21—C22—C23	118.1 (3)
C27—N7—C29	121.1 (5)	C21—C22—H22	121.0
C28—N7—C29	120.5 (5)	C23—C22—H22	121.0
C2—C1—O1	111.8 (3)	C22—C23—C24	120.9 (3)
C2—C1—H1	124.1	C22—C23—H23	119.6
O1—C1—H1	124.1	C24—C23—H23	119.6
C1—C2—C3	106.0 (3)	C25—C24—C23	121.5 (3)
C1—C2—H2	127.0	C25—C24—H24	119.2
C3—C2—H2	127.0	C23—C24—H24	119.2
C4—C3—C2	106.9 (3)	C26—C25—C24	117.4 (3)
C4—C3—H3A	126.5	C26—C25—H25	121.3
C2—C3—H3A	126.5	C24—C25—H25	121.3
C3—C4—O1	110.3 (2)	C25—C26—C21	121.9 (3)
C3—C4—C5	133.2 (3)	C25—C26—N6	128.5 (3)
O1—C4—C5	116.5 (2)	C21—C26—N6	109.6 (2)
O2—C5—N1	126.0 (2)	O7—C27—N7	123.8 (5)
O2—C5—C4	120.4 (2)	O7—C27—H27	118.1
N1—C5—C4	113.6 (2)	N7—C27—H27	118.1
O3—C6—N3	128.2 (3)	N7—C28—H28A	109.5
O3—C6—C7	125.2 (2)	N7—C28—H28B	109.5
N3—C6—C7	106.6 (2)	H28A—C28—H28B	109.5
N2—C7—C8	138.1 (3)	N7—C28—H28C	109.5
N2—C7—C6	113.7 (2)	H28A—C28—H28C	109.5
C8—C7—C6	108.2 (2)	H28B—C28—H28C	109.5
C9—C8—C13	120.6 (3)	N7—C29—H29A	109.5
C9—C8—C7	134.3 (3)	N7—C29—H29B	109.5
C13—C8—C7	105.0 (2)	H29A—C29—H29B	109.5
C8—C9—C10	117.8 (3)	N7—C29—H29C	109.5
C8—C9—H9	121.1	H29A—C29—H29C	109.5
C10—C9—H9	121.1	H29B—C29—H29C	109.5
			
N2—Mn1—O2—C5	2.65 (17)	O3—C6—C7—C8	179.1 (2)
N5—Mn1—O2—C5	175.17 (17)	N3—C6—C7—C8	0.2 (3)
O5—Mn1—O2—C5	−108.46 (18)	N2—C7—C8—C9	0.4 (5)
O3—Mn1—O2—C5	3.8 (3)	C6—C7—C8—C9	−178.8 (3)
O6—Mn1—O2—C5	97.10 (18)	N2—C7—C8—C13	179.3 (3)
N2—Mn1—O3—C6	3.01 (16)	C6—C7—C8—C13	0.0 (3)
N5—Mn1—O3—C6	−169.32 (16)	C13—C8—C9—C10	−0.5 (4)
O5—Mn1—O3—C6	114.71 (16)	C7—C8—C9—C10	178.2 (3)
O2—Mn1—O3—C6	1.9 (3)	C8—C9—C10—C11	1.2 (5)
O6—Mn1—O3—C6	−92.44 (17)	C9—C10—C11—C12	−1.2 (5)
N2—Mn1—O5—C18	−169.67 (16)	C10—C11—C12—C13	0.4 (5)
N5—Mn1—O5—C18	6.30 (17)	C11—C12—C13—C8	0.2 (4)
O2—Mn1—O5—C18	−92.60 (17)	C11—C12—C13—N3	−178.6 (3)
O3—Mn1—O5—C18	111.36 (17)	C9—C8—C13—C12	−0.2 (4)
O6—Mn1—O5—C18	17.1 (3)	C7—C8—C13—C12	−179.2 (2)
N2—Mn1—O6—C19	172.82 (18)	C9—C8—C13—N3	178.8 (2)
N5—Mn1—O6—C19	−2.79 (17)	C7—C8—C13—N3	−0.2 (3)
O5—Mn1—O6—C19	−13.5 (3)	C6—N3—C13—C12	179.3 (3)
O2—Mn1—O6—C19	96.97 (18)	C6—N3—C13—C8	0.3 (3)
O3—Mn1—O6—C19	−108.96 (18)	C17—O4—C14—C15	0.3 (4)
C5—N1—N2—C7	−177.6 (2)	O4—C14—C15—C16	−0.3 (4)
C5—N1—N2—Mn1	−0.9 (3)	C14—C15—C16—C17	0.2 (4)
O5—Mn1—N2—C7	−92.86 (19)	C15—C16—C17—O4	0.0 (3)
O2—Mn1—N2—C7	175.9 (2)	C15—C16—C17—C18	−177.2 (3)
O3—Mn1—N2—C7	−3.56 (18)	C14—O4—C17—C16	−0.1 (3)
O6—Mn1—N2—C7	84.04 (19)	C14—O4—C17—C18	177.5 (2)
O5—Mn1—N2—N1	90.30 (18)	Mn1—O5—C18—N4	−5.7 (3)
O2—Mn1—N2—N1	−0.89 (17)	Mn1—O5—C18—C17	174.99 (18)
O3—Mn1—N2—N1	179.61 (18)	N5—N4—C18—O5	0.0 (3)
O6—Mn1—N2—N1	−92.79 (18)	N5—N4—C18—C17	179.4 (2)
C18—N4—N5—C20	−179.0 (2)	C16—C17—C18—O5	177.8 (3)
C18—N4—N5—Mn1	6.2 (2)	O4—C17—C18—O5	0.9 (4)
O5—Mn1—N5—C20	177.8 (2)	C16—C17—C18—N4	−1.6 (4)
O2—Mn1—N5—C20	−88.14 (19)	O4—C17—C18—N4	−178.5 (2)
O3—Mn1—N5—C20	87.97 (19)	Mn1—O6—C19—N6	−175.7 (2)
O6—Mn1—N5—C20	2.83 (18)	Mn1—O6—C19—C20	2.7 (3)
O5—Mn1—N5—N4	−7.14 (16)	C26—N6—C19—O6	178.8 (3)
O2—Mn1—N5—N4	86.91 (18)	C26—N6—C19—C20	0.2 (3)
O3—Mn1—N5—N4	−96.98 (17)	N4—N5—C20—C21	0.2 (5)
O6—Mn1—N5—N4	177.88 (18)	Mn1—N5—C20—C21	175.1 (3)
C4—O1—C1—C2	−0.1 (4)	N4—N5—C20—C19	−177.3 (2)
O1—C1—C2—C3	0.0 (4)	Mn1—N5—C20—C19	−2.4 (3)
C1—C2—C3—C4	0.0 (4)	O6—C19—C20—N5	−0.6 (4)
C2—C3—C4—O1	−0.1 (3)	N6—C19—C20—N5	178.1 (2)
C2—C3—C4—C5	179.2 (3)	O6—C19—C20—C21	−178.9 (3)
C1—O1—C4—C3	0.1 (3)	N6—C19—C20—C21	−0.2 (3)
C1—O1—C4—C5	−179.3 (3)	N5—C20—C21—C22	2.7 (6)
Mn1—O2—C5—N1	−4.7 (3)	C19—C20—C21—C22	−179.7 (3)
Mn1—O2—C5—C4	176.15 (18)	N5—C20—C21—C26	−177.5 (3)
N2—N1—C5—O2	3.9 (3)	C19—C20—C21—C26	0.1 (3)
N2—N1—C5—C4	−176.9 (2)	C26—C21—C22—C23	−0.5 (4)
C3—C4—C5—O2	−172.0 (3)	C20—C21—C22—C23	179.3 (3)
O1—C4—C5—O2	7.2 (4)	C21—C22—C23—C24	0.9 (4)
C3—C4—C5—N1	8.7 (4)	C22—C23—C24—C25	−0.6 (5)
O1—C4—C5—N1	−172.1 (2)	C23—C24—C25—C26	−0.1 (5)
Mn1—O3—C6—N3	176.3 (2)	C24—C25—C26—C21	0.5 (4)
Mn1—O3—C6—C7	−2.4 (3)	C24—C25—C26—N6	−179.5 (3)
C13—N3—C6—O3	−179.2 (3)	C22—C21—C26—C25	−0.2 (4)
C13—N3—C6—C7	−0.3 (3)	C20—C21—C26—C25	180.0 (2)
N1—N2—C7—C8	1.0 (5)	C22—C21—C26—N6	179.8 (2)
Mn1—N2—C7—C8	−175.8 (3)	C20—C21—C26—N6	0.0 (3)
N1—N2—C7—C6	−179.9 (2)	C19—N6—C26—C25	179.9 (3)
Mn1—N2—C7—C6	3.4 (3)	C19—N6—C26—C21	−0.1 (3)
O3—C6—C7—N2	−0.3 (4)	C28—N7—C27—O7	2.2 (7)
N3—C6—C7—N2	−179.3 (2)	C29—N7—C27—O7	−178.0 (5)

### Hydrogen-bond geometry (Å, °)

**Table:**

*D*—H···*A*	*D*—H	H···*A*	*D*···*A*	*D*—H···*A*
O1W—H1W1···O3	0.86 (1)	2.06 (2)	2.896 (3)	165 (4)
O1W—H1W2···O7	0.86 (1)	1.93 (2)	2.762 (5)	161 (4)
N3—H3···O1W^i^	0.86 (1)	1.96 (1)	2.811 (4)	176 (3)
N6—H6···O6^ii^	0.87 (1)	2.05 (1)	2.899 (3)	167 (3)

Symmetry codes: (i) −*x*, −*y*, −*z*+1; (ii) −*x*+1, −*y*+1, −*z*+1.
